# Pesticide exposure and rhinitis: A cross-sectional study among farmers in Pitsanulok, Thailand

**DOI:** 10.12688/f1000research.53257.1

**Published:** 2021-06-15

**Authors:** Yuwayong Juntarawijit, Chudchawal Juntarawijit

**Affiliations:** 1 Faculty of Nursing, Naresuan University, Phitsanulok, 65000, Thailand; 2 Faculty of Agriculture, Natural Resources and Environment, Naresuan University, Phitsanulok, 65000, Thailand

**Keywords:** pesticide exposure, insecticide effect, herbicide effect, fungicide effect, rhinitis, farmer health

## Abstract

**Background**: Pesticide exposure has been suspected to cause rhinitis, a common disease that affects the health and wellbeing of millions of people around the world. The objective of this cross-sectional study was to examine the association between pesticide use and rhinitis prevalence among farmers in Phitsanulok province, Thailand.

**Methods**: Data on pesticide use and rhinitis use were collected by an in-person interview questionnaire. Data from 9,649 participants were included in the analysis. The association between pesticide exposure and rhinitis was determined by multiple variable logistic regression, adjusted for potential confounding factors.

**Results**: It was found that 6.3% of study participants had rhinitis, which was defined as having one or more of the following symptoms: sneezing, runny nose, and nasal congestion, within the past year. The study found each type of pesticide (insecticides, herbicides, fungicides, rodenticides, molluscicides) to be significantly associated with rhinitis, with the odds ratio ranging from 1.67 (95% CI 1.41-1.99) for the fungicide group to 7.19 (95% CI 4.67-11.06) for insecticide. For individual pesticides, the study found 33 out of 35 to be significantly associated with rhinitis with the OR values ranging from 1.30 (95% CI 1.00-1.69) for alachlor to 5.67 (95% CI 3.16-10.16) for Bordeaux mixture.

**Conclusions**: This study found a prevalence of rhinitis among farmers in Phitsanulok, Thailand to be associated with the long-term exposure of many individual pesticides. This finding was consistent with other literature. Effects of pesticide on rhinitis should receive more attention from the public health and other organizations responsible for the farmers’ health.

## Background

Rhinitis is a common disease that affects general health, and quality of life. Approximately, 10% to 30% of the different populations worldwide suffer from this disease ^1^. One study in Bangkok, Thailand, has reported a prevalence of chronic rhinitis to be approximately 13% ^2^. In clinical terms, rhinitis refers to the inflammatory disease of the nasal mucosa, which can cause the following symptoms: nasal congestion, rhinorrhea, and sneezing ^3^. Although rhinitis does not have a strict classification criterion, it can be classified into allergic rhinitis (AR), and nonallergic rhinitis (NAR). Both have the same nasal symptoms, with the difference that AR is triggered by allergens. Several factors can trigger NAR, including cold air, climate change, cooking smells, chemical odour, cigarette smoke, volatile organic chemicals, exercise, alcohol ingestion ^4^, and cooking fumes ^5^. NAR can be further classified by their pathological mechanisms into several subtypes, including occupation rhinitis, hormonal rhinitis, drug-induced rhinitis, food-induced rhinitis, emotion-induced rhinitis, etc ^6^. Approximately 43% of all rhinitis cases are AR, and 23% are NAR, while 34% of the cases are a mixture of both ^4^.

Studies found pesticide exposure to increase the risk of several respiratory problems, e.g., asthma, chronic bronchitis ^7^, and rhinitis ^8^. One study among grape farmers in Greece reported a higher prevalence of AR among those who used pesticides ^9^. Another study in France found that children living in areas surrounding vineyards had a higher rate of rhinitis symptoms (OR=3.56; 95% CI 1.04–12.12) ^10^. In an occupational setting, a study found number of hours working in the greenhouse per day to be associated with rhinitis (OR, 1.85; 95% CI, 1.05–3.23) ^11^. A large survey of farmworkers in the United States of America (U.S.A) found that insecticide and herbicide use has significantly increased the risk of allergic rhinitis and asthma ^12^.

Currently, studies on the effects of individual pesticides are limited. Pesticides that were found to have a positive association with rhinitis were bipyridyl herbicides such as paraquat, and the broad-spectrum herbicides 2,4-D, glyphosate, dithiocarbamate fungicides including benomyl, and insecticide diazinon ^9, 13, 14^. In one study, pesticides, especially cholinesterase inhibitors, e.g., organophosphates and carbamates pesticides, were found to cause neurogenic inflammation and muscarinic effects of the respiratory airway, as well as modification of the inflammatory response to other allergens ^15^.

The main objective of this cross-sectional study was to determine the association between pesticide use and rhinitis among farmers in Phitsanulok, Thailand. The use of a large sample size in this study has provided the opportunity to assess also the risks that different groups and subgroups of pesticide exposure have had on these individuals. The study results would be useful for disease prevention, and comparison with other studies.

## Methods

### Study design and setting

This study was a cross-sectional study. Participants were farmers in Phitsanulok province, located about 370 km north of Bangkok, Thailand. In 2019, the province had approximately 865,368 people (342,787 households) from nine districts. The major crops in the province are rice, sugarcane, and maize ^16, 17^.

### Study participants and sampling procedure

Multistage sampling was used for the random selection of the participants. The three districts were randomly selected from nine districts in the Phitsanulok province. From all the selected districts, 18 out of 26 (69.2%) sub-districts were further selected. In each sub-district, all local hospitals participated in the study and provided support for data collection. In each sub-district, farmers were selected by village health volunteers (VHV), who were working in the hospitals. The data from the local authority and personal contacts were used by the VHV for selecting the farmers. Sample size (n=9,649) was achieved with the snowball sampling technique. From each family, one adult aged 20 years or older who does agricultural work, was interviewed.

The minimum sample size was calculated to be 10,002, based on the following assumptions: significance level = 95%; power of detection = 80%; ratio of unexposed/exposed = 1; percent of unexposed with outcome = 10% ^2^; odds ratio = 1.2.

### Study questionnaire and data collection

Data were collected by using an in-person interview questionnaire (provided as Extended data in English and Thai) ^18^. Data on rhinitis was collected by using a modified form of SFAR (Score For Allergic Rhinitis) questionnaire which is recommended for population studies, where medical diagnosis and objective measurements were absent or difficult to obtain ^19^. Self-reported rhinitis was defined as the participant answering “yes” to the question: “during the last 12 months, have you ever had symptoms such as sneezing, or a runny, or blocked nose when you did not have a cold or the flu?”. Allergic rhinitis was also determined on a “yes” response to the question: “Have you ever been told by a doctor that you had allergic rhinitis?”.

For pesticide exposure, the data was collected by a questionnaire used in our previous study ^20^. Data on the long-term use of pesticides, either by types of pesticides (insecticide, herbicide, fungicide, rodenticide, and molluscicide), or by specific individual pesticides, were collected. A list of 35 individual pesticides were chosen from those that were commonly used in Thailand and were reported to cause adverse health effects ^20, 21^. Participants were asked whether they have ever used pesticides, defined as a mixture, or spray pesticides, in their lifetime. Participants were also asked to provide data on the duration (days/year and total years) of pesticide use. This information was used to calculate total days, and quartiles of days using each pesticide in the farmers’ lifetime.

Lifetime pesticide use measured in days = [Number of days per year] × [total years]

Data was collected from October 2020 to February 2021, by 210 VHV. Before data collection, these volunteers had to attend a one-day training program to be informed on the purpose of the study and to learn how to properly interview and collect data by using an online questionnaire. The interview mostly took place in the participant’s home. However, sometimes it was done in a local temple or hospital.

### Statistical analysis

Demographic data were analysed using descriptive statistics. Comparison of categorical data was analysed using the Chi-square test. The association between pesticide exposure and rhinitis prevalence was analysed using multivariable logistic regression, and both crude and adjusted odds ratios (OR) and 95% confidence interval (CI) were reported. Adjusted variables were gender (male, female), age (continuous), marital status (married, single, divorced/widow/separated), education (non-educated, primary school, secondary school, college degree or higher), family income (<5000 THB, 5001-10000 THB, 10001-30000THB, > 30000 THB), cigarette smoking (non-smoker, ex-smoker, current smoker), alcohol consumption (non- drinker, ex- drinker, regular- drinker). Data analysis was performed using IBM SPSS version 26, and OpenEpi (online version 3.01). All statistical values were two-tailed, and a p-value < 0.05 was considered as statistically significant.

### Ethical considerations

The study was approved by the Ethical Committee of Naresuan University (COA No. 657/2019) and written informed consent from the participants was obtained before the interview process. 

## Results

It was found that the proportion of female participants was slightly higher than that of the male participants ( Table 1 and the underlying data ^22^). Most of these individuals were aged 40 years and older with an average age of 55 (± 12 years), married (78.0%), finished primary school or with lower education (77.2), and had an average family income of 10,000 THB or less. A total of 8.7% of these females are cigarette smokers, and 13.7% consume alcohol.

**Table 1.** Demographic data of study participants.

Characteristics	Number (%), N = 9649
Gender	
Male	4163 (43.1)
Female	5486 (56.9)
Age, years	
20–30	306 (3.2)
31–40	840 (8.7)
41–50	2000 (20.7)
51–60	3174 (32.9)
61+	3329 (34.5)
Mean ± SD = 55 ± 12	
Min-Max = 20-92	
Marital status	
Married	7523 (78.0)
Single	737 (7.6)
Divorce/widow/separated	1389 (14.4)
Education	
Non-educated	402 (4.2)
Primary school	7046 (73.0)
Secondary school	2063 (21.4)
College degree or higher	138 (1.4)
Family income, THB/month	
<5000	3168 (32.8)
5001–10000	5739 (59.5)
10001–30000	668 (6.9)
>30000	74 (0.8)
Cigarette smoking	
Non-smoker	8511 (88.2)
Ex-smoker	303 (3.1)
Current smoker	835 (8.7)
Alcohol consumption	
Non-drinker	7865 (81.5)
Ex-drinker	461 (4.8)
Regular-drinker	1323 (13.7)

The prevalence of rhinitis was found to be 6.3% (609/9649) ( Table 2). Of the three symptoms, sneezing was found to be the most common (4.0%), followed by nasal congestion (3.0%), and runny nose (2.9%). Only 16.3% had eye irritations together with rhinitis symptoms. The months with the highest frequency of symptoms were March to July (summer season in Thailand), and November to February (winter season). The prevalence of rhinitis was significantly associated with marital status, education, family income, cigarette smoking, and alcohol consumption ( Table 3). 

**Table 2.** Prevalence of rhinitis and its characteristics.

	N (%), N=9649
Having symptoms in the past year	
Sneezing	390 (4.0)
Stuffy nose	294 (3.0)
Runny nose	282 (2.9)
Rhinitis (having one of the above three symptoms)	609 (6.3)
Having eyes irritation while having rhinitis	99 (16.3)
Month of having the symptoms (n=609)	
January	177 (29.3)
February	103 (17.0)
March	76 (12.6)
April	100 (16.5)
May	258 (42.6)
June	272 (44.9)
July	245 (40.5)
August	95 (15.7)
September	61 (10.1)
October	53 (8.8)
November	99 (16.4)
December	137 (22.6)
Allergens activated the symptoms (n=609)	
Dust	467 (76.7)
Mites	282 (46.3)
Smoke	275 (45.2)
Cooking fume	190 (31.4)
Straw or grass	101 (16.6)
Pet	33 (5.4)
Pollen	34 (5.6)
Patients who believed they have allergy	104 (17.1)
Patients that had allergic testing	36 (5.9)
Patients with positive result	24 (3.9)
Patients who were diagnosed with asthma or allergy	39 (6.7)

**Table 3.** Association between demographic data and rhinitis prevalence.

	Not rhinitis	Rhinitis	P-value ^a^
Gender			0.176
Male	3884 (93.3)	279 (6.7)	
Female	5156 (94.0)	330 (6.0)	
Age			0.488
20–30	285 (93.1)	21 (6.9)	
31–40	777 (92.5)	63 (7.5)	
41–50	1867 (93.4)	133 (6.7)	
51–60	2983 (94.0)	191 (6.0)	
>60	3128 (94.0)	201 (6.0)	
Marital status			0.010 *
Married	7021 (93.3)	502 (6.7)	
Single	693 (94.0)	44 (6.0)	
Divorce/widow/separated	1326 (95.5)	63 (4.5)	
Education complete			<0.001 *
Non-educated	381 (94.8)	21 (5.2)	
Primary school	6655 (94.5)	391 (5.5)	
Secondary school	1877 (91.0)	186 (9.0)	
College degree or higher	127 (92.0)	11 (8.0)	
Family income, THB/month			<0.001 *
<5000	3067 (96.8)	101 (3.2)	
5001–10000	5330 (92.9)	409 (7.1)	
10001–30000	576 (86.2)	92 (13.8)	
>30000	67 (90.5)	7 (9.5)	
Cigarette smoking			0.001 *
Non-smoker	7997 (94.0)	514 (6.0)	
Ex-smoker	269 (88.8)	34 (11.2)	
Current smoker	774 (92.7)	61 (7.3)	
Alcohol consumption			<0.001 *
Non-drinker	7415 (94.3)	450 (5.7)	
Ex-drinker	406 (88.1)	55 (11.9)	
Regular-drinker	1219 (92.1)	104 (7.9)	

^a^ Chi-square test. * Statistically significant difference with p value <0.05

In this study, among the five groups of pesticides all were significantly associated with rhinitis. The OR values ranged between 1.67 (95%CI 1.41-1.99) for fungicide and 7.19 (95%CI 4.67-11.06) for insecticide ( Table 4).

**Table 4.** The associations between type of pesticides use and rhinitis prevalence.

Pesticide	Not rhinitis	Rhinitis	OR (crude)	OR (adjusted) ^a^
Any pesticide				
No	463 (96.5)	17 (3.5)	1.0	1.0
Yes	8575 (93.5)	592 (6.5)	**1.88 (1.15-3.07)** ^b^	**1.79 (1.09-2.94)**
Insecticide				
No	1996 (98.9)	22 (1.1)	1.0	1.0
Yes	7044 (92.3)	587 (7.7)	**7.56 (4.93-11.61)**	**7.19 (4.67-11.06)**
Q1	1837 (95.1)	94 (4.9)	1.0	1.0
Q2	2009 (93.7)	136 (6.3)	**1.32 (1.01-1.73)**	**1.36 (1.03-1.79)**
Q3	2048 (90.5)	215(9.5)	**2.05 (1.60-2.64)**	**2.18 (1.68-2.82)**
Q4	1150 (89.0)	142 (11.0)	**2.41 (1.84-3.16)**	**2.65 (2.00-3.52)**
P for trend				0.206
Herbicide				
No	1254 (98.4)	20 (1.6)	1.0	1.0
Yes	7786 (93.0)	586 (7.0)	**4.74 (3.03-7.44)**	**4.25 (2.71-6.69)**
Q1	2227 (95.2)	112 (4.8)	1.0	1.0
Q2	1864 (93.9)	122 (6.1)	**1.30 (1.00-1.69)**	**1.34 (1.03-1.76)**
Q3	2555 (92.0)	221 (8.0)	**1.72 (1.36-2.17)**	**1.77 (1.38-2.25)**
Q4	1140 (89.5)	134 (10.5)	**2.34 (1.80-3.03)**	**2.55 (1.94-3.36)**
P for trend				0.095
Fungicide				
No	5107 (95.2)	256 (4.8)	1.0	1.0
Yes	3933 (91.8)	353 (8.2)	**1.79 (1.52-2.11)**	**1.67 (1.41-1.99)**
Q1	1006 (93.7)	68 (6.3)	1.0	1.0
Q2	1020 (92.3)	100 (7.7)	1.23 (0.89-1.69)	1.23 (0.89-1.70)
Q3	766 (90.7)	79 (9.3)	**1.53 (1.09-2.14)**	**1.49 (1.05-2.11)**
Q4	959 (90.0)	106 (10.0)	**1.64 (1.19-2.25)**	**1.53 (1.10-2.13)**
P for trend				0.391
Rodenticide				
No	7371 (95.1)	379 (4.9)	1.0	1.0
Yes	1669 (87.9)	230 (12.1)	**2.68 (2.26-3.18)**	**2.63 (2.20-3.14)**
Q1	514 (88.9)	64 (11.1)	1.0	1.0
Q2	319 (83.5)	63 (16.5)	**1.59 (1.09-2.31)**	1.47 (0.99-2.17)
Q3	448 (90.3)	48 (9.7)	0.86 (0.58-1.28)	0.79 (0.53-1.19)
Q4	388 (87.6)	55 (12.4)	1.14 (0.78-1.67)	0.95 (0.64-1.43)
P for trend				0.758
Molluscicide				
No	7096 (95.0)	371 (5.0)	1.0	1.0
Yes	1944 (889.1)	238 (10.9)	**2.34 (1.98-2.78)**	**2.29 (1.92-2.73)**
Q1	573 (86.6)	89 (13.4)	1.0	1.0
Q2	404 (87.6)	57 (12.4)	0.91 (0.64-1.30)	0.90 (0.62-1.30)
Q3	544 (92.5)	44 (7.5)	0.52 (0.36-0.76)	0.50 (0.34-0.73)
Q4	423 (89.8)	48 (10.2)	0.73 (0.50-1.06)	0.67 (0.45-0.98)
P for trend				0.687

^a^ Adjusted variables were gender (male, female), age (continuous), marital status (married, single, divorce/willow/separated), education (non-educated, primary school, secondary school, college degree or higher), family income (<5000 THB, 5001-10000 THB, 10001-30000 THB, >30000 THB), cigarette smoking (non-smoker, ex-smoker, current smoker), alcohol consumption (non-drinker, ex-drinker, regular-drinker). ^b^ Significant OR were indicated in bold numbers.

For individual pesticides, the study found 33 out of 35 to be significantly associated with rhinitis, including six herbicides [glyphosate, paraquat, 2,4-Dichlorophenoxyacetic acid butachlor (2,4-D), butachlor, alachlor, and diuron], nine organophosphates (OP) insecticides [chlorpyrifos, folidol (parathion), methamidophos, monocrotophos, mevinphos, dicrotophos, dichlorvos, *O*-Ethyl *O*-(4-nitrophenyl) phenylphosphonothioate (EPN), and imidacloprid], four carbamate insecticides [carbaryl, methomyl, carbosulfan, and carbofuran], one pyrethroid insecticides – permethrin, six OC insecticides [endosulfan, dieldrin, aldrin, Dichlorodiphenyltrichloroethane (DDT), chlordane and heptachlor], and seven fungicides [metalaxyl, mancozeb, propineb, carbendazim, thiophanate, benomyl, and bordeaux mixture] ( Table 5).

**Table 5.** The associations between individual pesticide and rhinitis prevalence.

	Not rhinitis	Rhinitis	OR (Crude)	OR (Adjusted) ^a^
**Herbicides**				
**Glyphosate**				
No	1104 (97.3)	31 (2.7)	1.0	1.0
Yes	7936 (93.2)	578 (6.8)	**2.59 (1.80-3.74)** ^b^	**2.43 (1.68-3.52)**
Q1	2463 (94.6)	141 (5.4)	1.0	1.0
Q2	1853 (93.5)	129 (6.5)	1.22 (0.95-1.56)	1.29 (0.99-1.66)
Q3	1869 (91.6)	171 (8.4)	**1.60 (1.27-2.01)**	**1.67 (1.32-2.12)**
Q4	1751 (92.7)	137 (7.3)	**1.36 (1.07-1.74)**	**1.46 (1.13-1.89)**
P for trend				0.708
**Paraquat**				
No	3503 (95.6)	160 (4.4)	1.0	1.0
Yes	5537 (92.5)	449 (7.5)	**1.78 (1.48-2.14)**	**1.54 (1.28-1.87)**
Q1	1723 (93.4)	121 (6.6)	1.0	1.0
Q2	1233 (93.1)	91 (6.9)	1.05 (0.79-1.39)	1.10 (0.83-1.47)
Q3	1309 (90.4)	139 (9.6)	1.51 (1.17-1.95)	1.60 (1.23-2.08)
Q4	1272 (92.8)	98 (7.2)	1.10 (0.83-1.45)	1.16 (0.87-1.55)
P for trend				0.909
**2,4-D**				
No	4413 (94.9)	239 (5.1)	1.0	1.0
Yes	4627 (92.6)	370 (7.4)	**1.48 (1.25-1.75)**	**1.38 (1.61-1.63)**
Q1	1301 (92.3)	109 (7.7)	1.0	1.0
Q2	1161 (91.5)	108 (8.5)	1.11 (0.84-1.47)	1.09 (0.81-1.44)
Q3	1077 (92.4)	89 (7.6)	0.99 (0.74-1.32)	0.99 (0.72-1.33)
Q4	1089 (94.4)	64 (5.6)	0.70 (0.51-0.97)	0.71 (0.51-1.00)
**Butachlor**				
No	6868 (94.8)	374 (5.2)	1.0	1.0
Yes	2172 (90.2)	235 (9.8)	**1.99 (1.68-2.36)**	**1.82 (1.52-2.17)**
Q1	577 (90.3)	62 (9.7)	1.0	1.0
Q2	635 (90.1)	70 (9.9)	1.03 (0.72-1.47)	1.00 (0.69-1.45)
Q3	525 (89.8)	59 (10.1)	1.05 (0.72-1.52)	0.96 (0.65-1.42)
Q4	435 (90.8)	44 (9.2)	0.94 (0.63-1.41)	0.85 (0.55-1.30)
**Alachlor**				
No	8222 (93.9)	536 (6.1)	1.0	1.0
Yes	818 (91.8)	73 (8.2)	**1.37 (1.06-1.77)**	**1.30 (1.00-1.69)**
Q1	259 (92.8)	20 (7.2)	1.0	1.0
Q2	147 (87.5)	21 (12.5)	1.85 (0.97-3.53)	1.76 (0.89-3.51)
Q3	302 (95.3)	15 (4.7)	0.64 (0.32-1.28)	0.61 (0.29-1.28)
Q4	110 (86.6)	17 (13.4)	**2.00 (1.01-3.97)**	1.73 (0.81-3.67)
P for trend				0.492
**Diuron**				
No	8931 (93.8)	593 (6.2)	1.0	1.0
Yes	109 (87.2)	16 (12.8)	**2.21 (1.30-3.76)**	**1.73 (1.00-2.99)**
Q1	38 (97.4)	1 (2.6)	1.0	1.0
Q2	30 (93.8)	2 (6.3)	2.533 (0.22-29.29)	5.62 (0.10-318.16)
Q3	21 (87.5)	3 (12.5)	5.43 (0.53-55.52)	20.38 (0.40-103.23)
Q4	20 (66.7)	10 (33.3)	19.00 (2.27-159.20)	13.59 (0.53-350.81)
P for trend				0.706
**Organophosphate insecticides**				
**Abamectin **				
No	3952 (93.2)	290 (6.8)	1.0	1.0
Yes	5088 (94.1)	319 (5.9)	0.85 (0.73-1.01)	0.82 (0.69-0.97)
Q1	1630 (94.2)	100 (5.8)	1.0	1.0
Q2	1108 (93.7)	75 (6.3)	1.10 (0.81-1.50)	1.11 (0.81-1.52)
Q3	1191 (92.5)	97 (7.5)	**1.33 (1.00-1.78)**	**1.35 (1.00-1.82)**
Q4	1153 (96.1)	47 (3.9)	0.66 (0.47-0.95)	0.723 (0.50-1.04)
**Chlorpyrifos**				
No	7015 (94.5)	408 (5.5)	1.0	1.0
Yes	2025 (91.0)	201 (9.0)	**1.71 (1.43-2.04)**	**1.53 (1.28-1.84)**
Q1	535 (90.8)	54 (9.2)	1.0	1.0
Q2	658 (91.3)	63 (8.7)	0.95 (0.65-1.39)	0.96 (0.65-1.42)
Q3	393 (89.5)	46 (10.5)	1.16 (0.77-1.76)	1.22 (0.79-1.87)
Q4	439 (92.0)	38 (8.0)	0.86 (0.56-1.32)	0.84 (0.53-1.32)
P for trend				0.324
**Folidol/parathion**				
No	8017 (95.3)	391 (4.7)	1.0	1.0
Yes	1023 (82.4)	218 (17.6)	**4.37 (3.66-5.22)**	**3.95 (3.29-4.74)**
Q1	298 (89.2)	36 (10.8)	1.0	1.0
Q2	277 (86.8)	42 (13.2)	1.26 (0.78-2.02)	1.43 (0.87-2.35)
Q3	227 (79.4)	59 (20.6)	**2.15 (1.37-3.37)**	**2.19 (1.37-3.50)**
Q4	221 (73.2)	81 (26.8)	**3.03 (1.98-4.66)**	**2.93 (1.85-4.65)**
P for trend				0.157
**Methamidophos**				
No	8658 (94.0)	553 (6.0)	1.0	1.0
Yes	382 (87.2)	56 (12.8)	**2.30 (1.71-3.08)**	**2.06 (1.522-2.79)**
Q1	319 (89.6)	37 (10.4)	1.0	1.0
Q2	58 (87.9)	8 (12.1)	1.19 (0.53-2.68)	0.73 (0.29-1.81)
Q3	4 (50.0)	4 (50.0)	8.62 (2.07)	**8.22 (1.30-52.01)**
Q4	0 (0.0)	7 (100.0)	NA	NA
**Monocrotophos**				
No	8856 (93.9)	575 (6.1)	1.0	1.0
Yes	184 (84.4)	34 (15.6)	**2.85 (1.96-4.13)**	**2.44 (1.65-3.61)**
Q1	52 (88.1)	7 (11.9)	1.0	1.0
Q2	49 (84.5)	9 (15.5)	1.36 (0.47-3.95)	1.12 (0.30-4.11)
Q3	57 (96.6)	2 (3.4)	0.26 (0.05-1.31)	0.20 (0.03-1.24)
Q4	26 (61.9)	16 (38.1)	**4.57 (1.67-12.49)**	2.14 (0.55-8.30)
P for trend				0.169
**Mevinphos**				
No	8937 (93.8)	591 (6.2)	1.0	1.0
Yes	103 (85.1)	18 (14.9)	**2.64 (1.59-4.39)**	**2.32 (1.36-3.94)**
Low	64 (91.4)	6 (8.6)	1.0	1.0
High	39 (76.5)	12 (23.5)	**3.28 (1.14-9.45)**	2.70 (0.64-11.32)
**Dicrotophos**				
No	8868 (93.8)	587 (6.2)	1.0	1.0
Yes	172 (88.7)	22 (11.3)	**1.93 (1.23-3.04)**	**1.70 (1.07-2.71)**
Low	99 (88.4)	13 (11.6)	1.0	1.0
High	73 (89.0)	9 (11.0)	0.94 (0.38-2.31)	0.70 (0.24-2.11
**Dichlorvos**				
No	8951 (93.8)	591 (6.2)	1.0	1.0
Yes	89 (83.2)	18 916.8)	**3.06 (1.83-5.12)**	**2.77 (1.63-4.70)**
Low	52 (91.2)	5 (8.8)	1.0	1.0
High	36 (73.5)	13 (26.5)	**3.76 (1.23-11.46)**	5.87 (0.90-38.10)
**EPN**				
No	8837 (94.2)	545 (5.8)	1.0	1.0
Yes	203 (76.0)	64 (24.0)	**5.11 (3.81-6.86)**	**4.15 (3.06-5.62)**
Q1	60 (74.1)	21 (25.9)	1.0	1.0
Q2	49 (87.5)	7 (12.5)	0.41 (0.16-1.04)	0.45 (0.16-1.25)
Q3	48 (75.0)	16 (25.0)	0.95 (0.45-2.02)	0.98 (0.42-2.30)
Q4	46 (69.7)	20 (30.3)	1.24 (0.63-2.56)	0.98 (0.42-2.28)
P for trend				0.677
**Imidacloprid**				
No	8683 (93.9)	561 (6.1)	1.0	1.0
Yes	357 (88.1)	48 (11.9)	**2.08 (1.52-2.85)**	**1.88 (1.36-2.61)**
Q1	88 (83.0)	18 (37.5)	1.0	1.0
Q2	103 (91.2)	10 (8.8)	0.78 (0.21-1.08)	0.40 (0.15-1.03)
Q3	82 (94.3)	5 (5.7)	0.30 (0.11-1.08)	0.31 (0.10-1.01)
Q4	84 (84.8)	15 (15.2)	0.87 (0.41-1.84)	1.07 (0.45-2.56)
P for trend				0.394
**Profenofos**				
No	8822 (93.7)	590 (6.3)	1.0	1.0
Yes	218 (92.0)	19 (8.0)	1.30 (0.81-2.10)	1.26 (0.78-2.06)
Low	112 (94.1)	7 (5.9)	1.0	1.0
High	106 (89.9)	12 (10.2)	1.30 (0.81-2.10)	1.29 (0.79-2.11)
**Carbamate insecticides**				
**Carbaryl/Sevin**				
No	8415 (94.3)	511 (5.7)	1.0	1.0
Yes	625 (86.4)	98 (13.6)	**2.58 (2.05-3.23)**	**2.31 (1.82-2.93)**
Q1	170 (89.9)	19 (10.1)	1.0	1.0
Q2	150 (86.2)	24 (13.8)	1.43 (0.75-2.72)	1.47 (0.74-2.93)
Q3	171 (88.1)	23 (11.9)	1.20 (0.63-2.30)	1.07 (0.54-2.12)
Q4	134 (80.7)	32 (19.3)	**2.14 (1.16-3.94)**	**2.23 (1.31-4.39)**
P for trend				0.070
**Methomyl**				
No	8581 (94.1)	542 (5.9)	1.0	1.0
Yes	459 (87.3)	67 (12.7)	**2.31 (1.76-3.03)**	**2.02 (1.53-2.68)**
Q1	120 (81.1)	28 (18.9)	1.0	1.0
Q2	107 (91.5)	10 (8.5)	0.40 (0.19-0.86)	0.45 (0.20-1.01)
Q3	131 (87.9)	18 (12.1)	0.59 (0.31-1.12)	0.63 (0.32-1.24)
Q4	101 (90.2)	11 (9.8)	0.47 (0.22-0.98)	0.49 (0.22-1.09)
P for trend				0.576
**Carbosulfan**				
No	7945 (94.4)	467 (5.6)	1.0	1.0
Yes	1095 (88.5)	142 (11.5)	**2.21 (1.81-2.69)**	**2.09 (1.70-2.56)**
Q1	130 (82.8)	27 (17.2)	1.0	1.0
Q2	165 (88.7)	21 (11.3)	0.61 (0.33-1.13)	0.62 (0.33-1.17)
Q3	399 (88.5)	52 (11.5)	0.63 (0.38-1.04)	0.61 90.36-1.04)
Q4	401 (90.5)	42 (9.5)	0.50 (0.20-0.85)	0.49 (0.28-0.85)
P for trend				0.410
**Carbofuran**				
No	8336 (94.4)	497 (5.6)	1.0	1.0
Yes	704 (86.3)	112 (13.7)	**2.67 (2.14-3.32)**	**2.34 (1.86-2.93)**
Q1	239 (83.9)	46 (16.1)	1.0	1.0
Q2	112 (86.8)	17 (13.2)	0.79 (0.43-1.44)	0.66 (0.35-1.26)
Q3	184 (86.4)	29 (13.6)	0.82 (0.50-1.35)	0.71 (0.41-1.22)
Q4	169 (89.4)	20 (10.6)	0.62 (0.35-1.08)	0.50 (0.27-0.90)
P for trend				0.296
**Pyrethroid insecticides**				
**Permethrin**				
No	8083 (94.0)	515 (6.0)	1.0	1.0
Yes	957 (91.1)	94 (8.9)	**1.54 (1.23-1.94)**	**1.60 (1.27-2.03)**
Q1	304 (88.1)	41 (11.9)	1.0	1.0
Q2	169 (91.4)	16 (8.6)	0.70 (0.38-1.29)	0.73 (0.38-1.40)
Q3	349 (92.6)	28 (7.4)	0.60 (0.36-0.99)	0.82 (0.47-1.43)
Q4	135 (93.8)	9 (6.3)	0.49 (0.23-1.05)	0.36 (0.16-0.82)
P for trend				0.084
**Organochlorine insecticides**				
**Endosulfan**				
No	7814 (94.6)	447 (5.4)	1.0	1.0
Yes	1226 (88.3)	162 (11.7)	**2.31 (1.91-2.79)**	**2.17 (1.78-2.64)**
Q1	378 (84.4)	70 (15.6)	1.0	1.0
Q2	300 (93.5)	21 (6.5)	0.38 (0.23-0.63)	0.36 (0.21-0.62)
Q3	261 (88.5)	34 (11.5)	0.70 (0.45-1.09)	0.60 (0.38-0.95)
Q4	287 (88.6)	37 (11.4)	0.70 (0.45-1.07)	0.60 (0.38-0.95)
P for trend				0.888
**Dieldrin**				
No	8979 (93.9)	585 (6.1)	1.0	1.0
Yes	61 (71.8)	24 (28.2)	**6.04 (3.74-9.76)**	**5.08 (3.07-8.43)**
Low	46 (90.2)	5 (9.8)	1.0	1.0
High	15 (44.1)	19 (55.9)	**11.65 (3.71-36.61)**	**9.23 (1.20-71.11)**
**Aldrin**				
No	8992 (93.9)	589 (6.1)	1.0	1.0
Yes	48 (70.6)	20 (29.4)	**6.36 (3.75-10.79)**	**4.83 (2.78-8.39)**
Low	33 (89.2)	4 (10.8)	1.0	1.0
High	15 (48.4)	16 (51.6)	**8.80 (2.51-30.84)**	NA
**DDT**				
No	8759 (94.4)	524 (5.6)	1.0	1.0
Yes	281 (76.8)	85 (23.2)	**5.05 (3.91-6.55)**	**4.57 (3.43-5.99)**
Q1	74 (78.7)	20 (21.3)	1.0	1.0
Q2	86 (78.9)	23 (21.1)	0.99 (0.50-1.94)	1.39 (0.64-2.98)
Q3	87 (83.7)	17 (16.3)	0.72 (0.35-1.48)	0.71 (0.32-1.58)
Q4	34 (57.6)	25 (42.4)	**2.72 (1.33-5.56)**	1.52 (0.67-3.47)
P for trend				0.372
**Chlordane**				
No	8948 (93.9)	586 (96.2)	1.0	1.0
Yes	92 (80.0)	23 (20.0)	**3.82 (2.40-6.07)**	**3.43 (2.11-5.58)**
Low	57 (87.7)	8 (12.3)	1.0	1.0
High	35 (70.0)	15 (30.0)	**3.05 (1.17-7.94)**	2.25 (0.48-10.63)
**Heptachlor**				
No	8941 (93.9)	584 (6.1)	1.0	1.0
Yes	99 (79.8)	25 (20.2)	**3.87 (2.47-6.04)**	**3.29 (2.07-5.24)**
Low	61 (91.0)	6 (9.0)	1.0	1.0
High	38 (66.7)	19 (33.3)	**5.08 (1.86-13.86)**	0.81 (0.10-6.84)
**Fungicide**				
**Metalaxyl**				
No	8315 (93.9)	544 (6.1)	1.0	1.0
Yes	725 (91.8)	65 (8.2)	**1.37 (1.05-1.79)**	**1.35 (1.03-1.78)**
Q1	210 (92.9)	16 (7.1)	1.0	1.0
Q2	186 (95.4)	9 (4.6)	0.64 (0.27-1.47)	0.53 (0.21-1.31)
Q3	205 (92.3)	17 (7.7)	1.09 (0.54-2.21)	0.87 (0.40-1.89)
Q4	124 (84.4)	23 (15.6)	**2.43 (1.24-4.78)**	1.71 (0.79-3.71)
P for trend				0.084
**Mancozeb**				
No	8407 (94.1)	525 (5.9)	1.0	1.0
Yes	633 (88.3)	84 (11.7)	**2.13 (1.67-2.71)**	**1.99 (1.55-2.55)**
Q1	166 (86.5)	26 (13.5)	1.0	1.0
Q2	182 (91.5)	17 (8.5)	0.60 (0.31-1.14)	0.57 (0.28-1.15)
Q3	138 (90.8)	14 (9.2)	0.65 (0.33-1.29)	0.53 (0.25-1.11)
Q4	147 (84.5)	27 (15.5)	1.17 (0.66-2.10)	0.90 (0.46-1.74)
P for trend				0.596
**Maneb**				
No	8625 (93.8)	575 (6.3)	1.0	1.0
Yes	415 (92.4)	34 (7.6)	1.23 (0.56-1.76)	1.24 (0.86-1.79)
Q1	109 (94.8)	6 (5.2)	1.0	1.0
Q2	113 (91.9)	10 (8.1)	1.61 (0.57-4.58)	1.90 (0.54-6.71)
Q3	101 (95.3)	5 (4.7)	0.90 (0.27-3.04)	0.76 (0.18-3.19)
Q4	92 (87.6)	13 (12.4)	2.57 (0.94-7.02)	1.04 (0.28-3.87)
P for trend				0.808
**Propineb**				
No	8624 (93.9)	560 (6.1)	1.0	1.0
Yes	398 (89.0)	49 (11.0)	**1.90 (1.40-2.59)**	**1.84 (1.34-2.53)**
Q1	125 (31.4)	9 (18.4)	1.0	1.0
Q2	80 (88.9)	10 (11.1)	1.74 (0.68-4.46)	1.98 (0.68-5.76)
Q3	109 (87.9)	15 (12.1)	1.91 (0.80-4.54)	1.97 (0.73-5.33)
Q4	84 (84.8)	15 (15.2)	**2.48 (1.04-5.93)**	2.51 (0.91-6.93)
P for trend				0.301
**Carbendazim**				
No	7856 (94.2)	484 (5.8)	1.0	1.0
Yes	1184 (90.5)	125 (9.5)	**1.71 (1.40-2.11)**	**1.57 (1.27-1.94)**
Q1	316 (88.0)	43 (12.0)	1.0	1.0
Q2	328 (91.6)	30 (8.4)	0.67 (0.41-1.10)	0.72 (0.43-1.19)
Q3	311 (89.6)	36 (10.4)	0.85 (0.53-1.36)	0.80 (0.49-1.31)
Q4	229 (93.5)	16 (6.5)	0.51 (0.28-0.93)	0.47 (0.25-0.870
P for trend				0.148
**Thiophanate**				
No	8828 (93.8)	582 (6.2)	1.0	1.0
Yes	212 (88.7)	27 (11.3)	**1.93 (1.28-2.91)**	**1.90 (1.25-2.90)**
Q1	52 (86.7)	8 (13.3)	1.0	1.0
Q2	60 (95.2)	3 (4.8)	0.33 (0.08-1.29)	0.22 (0.04-1.06)
Q3	53 (91.4)	5 (8.6)	0.61 (0.19-2.00)	0.66 (0.15-2.79)
Q4	47 (81.0)	11 (19.0)	1.52 (0.56-4.10)	1.16 (0.31-4.31)
P for trend				0.365
**Benomyl**				
No	8916 (93.8)	590 (6.2)	1.0	1.0
Yes	124 (86.7)	19 (13.3)	**2.32 (1.42-3.78)**	**2.03 (1.22-3.36)**
Low	72 (92.3)	6 (7.7)	1.0	1.0
High	52 (80.0)	13 (20.0)	**3.00 (1.07-8.41)**	1.28 (0.30-5.54)
**Bordeaux mixture**				
No	8996 (93.8)	591 (6.2)	1.0	1.0
Yes	44 (71.0)	18 (29.0)	**6.23 (34.58-10.84)**	**5.67 (3.16-10.16)**
Q1	13 (76.5)	4 (23.5)	1.0	1.0
Q2	14 (100.0)	0	NA	NA
Q3	14 (73.7)	5 (26.3)	1.61 (0.26-5.29)	0.63 (0.02-21.11)
Q4	3 (25.0)	9 (75.0)	**9.75 (1.74-54.53)**	0.62 (0-360.35)

^a^ Adjusted variables were gender (male, female), age (continuous), marital status (married, single, divorce/willow/separated), education (non-educated, primary school, secondary school, college degree or higher), family income (<5000 THB, 5001-10000 THB, 10001-30000 THB, >30000 THB), cigarette smoking (non-smoker, ex-smoker, current smoker), alcohol consumption (non-drinker, ex-drinker, regular-drinker). ^b^ Significant OR were indicated in bold numbers.

## Discussion

The results of this study indicate that 6.3% of the farmers in Phitsanulok province had rhinitis. This prevalence rate was much lower than those found in previous studies, such as the 67% of the farmers in the Agriculture Health Study (AHS) ^21^, 57% of farm residents in New York ^23^, and 31% of greenhouse flower and ornamental plant growers in Europe ^24^. Though it is difficult to differentiate between AR and NAR ^4^, the rhinitis among the study participants was more likely to be NAR because the study results found that only 16.3% of the participants had eye irritation, most of them reported having symptoms during the summer season (March-July), or the winter season (November-February) ( Table 2). Besides, only 104 farmers (17.1%) believed they have an allergy, and 24 (3.9%) had allergic test positive. These were consistent with the facts that NAR is a type of rhinitis that can be triggered by changes in the weather, without having any involvement from the immune system or allergens, or rarely exhibiting itchy eye symptoms ^25^.

In this study, the prevalence of rhinitis was significantly associated with a history of using several pesticides. Among five groups of pesticides surveyed in this study, insecticides, herbicides, fungicides, rodenticides, and molluscicides, all had a positive association with rhinitis, with the OR from 1.67 (95% CI 1.41-1.99) for the fungicide group to 7.19 (95% CI 4.67-11.06) for insecticide ( Table 4). Among the insecticide group, those individual pesticides that are associated with rhinitis were herbicides (glyphosate, paraquat, 2,4-D, butachlor, alachlor, and diuron), OP insecticide (chlorpyrifos, folidol (parathion), methamidophos, monocrotophos, mevinphos, dicrotophos, dichlorvos, EPN, and imidacloprid), carbamate insecticides (carbaryl, methomyl, carbosulfan, and carbofuran), pyrethroid insecticides (ermethrin; OC insecticides - endosulfan, dieldrin, aldrin, DDT, chlordane, and heptachlor), and fungicides (metalaxyl, mancozeb, propineb, carbendazim, thiophanate, benomyl, and bordeaux mixture) ( Table 5). This was consistent with the literature, although a small number of the studies have focused on pesticide use and its association with rhinitis. A previous study with grape farmers in Crete, Greece reported a higher prevalence of allergic rhinitis symptoms among herbicide users, especially glyphosate when compared to the control group (OR, 3.0; 95% CI, 1.4 to 6.2) ^9^. The study also observed that the herbicide paraquat and the insecticide carbamate can increase the risk of rhinitis. The AHS in the U.S.A has shown that the elevated risk of rhinitis was observed among those who were exposed to pesticides such as glyphosate, petroleum oil, 2,4-D, metolachlor ^26^, diazinon, carbofuran, malathion, and carbaryl ^21^. Pyrethroid insecticide exposure, however, has been reported to involve respiratory symptoms e.g., irritation of the respiratory tract, and nose or throat irritation accompanied by sneezing or coughing, as appose to rhinitis ^27^. Research on carbamate insecticides has found this type of insecticides to increase the risk of asthma, a disease closely related to rhinitis ^28^. In AHS, the fungicide captan was significantly associated with an increased number of rhinitis episodes (OR = 1.32; 95% CI = 1.17–1.49) among those with 7–12 symptoms per year) ^21^. Pesticide exposure has also been associated with some respiratory symptoms, including wheezing airway irritation, dry/sore throat, cough, breathlessness, and chest tightness ^27^.

One possible biomechanism that can explain this effect was that pesticides could increase susceptibility to allergens or other stimuli, by causing irritation, inflammation and immune suppression in the airway ^29^. In addition, pesticides can also modify inflammatory responses of other allergens ^30^. Pesticides and the solvent composition of the mixture that contains them are lipid-soluble, therefore could irritate the nose and throat when inhaled ^31^. OPs, and carbamate insecticides are cholinesterase inhibitors that can inhibit acetylcholinesterase and cause more secretion of mucus in the nose and airway system ^32^. Studies among the asthmatic group, found OP exposure to induce bronchospasm and airway hyperreactivity ^33^. In vitro study showed the cytotoxic effects of fungicides on bronchial epithelial cells ^34^. Evidence from both animal and human studies showed that herbicides can cause airway inflammation ^35^. A recent study found herbicide 2,4-D to increase the mast cells in the nasal epithelium ^36^.

 This study has some limitations. Information on other pollutant exposure, such as grain and hay handling, environmental pesticide exposure, and maintenance activities, e.g., repairing engines and pesticide equipment, were not collected and used to control for confounding bias. However, it is more likely that both the control and study groups will have a similar risk to those factors. A previous AHS study found that the association between pesticide exposure and rhinitis was independent of crop, animal, or farm maintenance exposures ^21^. In this study, self-reported rhinitis and not excluding other respiratory diseases could have resulted in the overestimation of rhinitis prevalence. However, this would have been unlikely to occur as just 6.3% of the farmers reported having rhinitis ( Table 2). Recall bias and reliability of pesticide exposure data might be another concern. However, a study found that the questionnaire and data collection technique used in the AHS which uses, a similar questionnaire and data collection technique, to be reliable and reproducible ^37^. If recall bias had occurred, it would have been equally distributed among the study and control groups, and it would minimize rather than increase the association ^38^. Moreover, the use of cross-sectional design for this study does not allow for the results to be interpreted in terms of cause-and-effect association.

To our knowledge, in Thailand, this was the first study that links pesticide exposure to rhinitis. The use of large sample size, and detailed data on pesticide exposure in the study has identified new pesticides along with the association of many individual pesticides to rhinitis.

## Conclusion

The prevalence of rhinitis among farmers in Phitsanulok province, Thailand was significantly associated with pesticide exposure, either by type or individual pesticide. This information should receive more public attention and be incorporated into the rhinitis prevention program. More studies should be conducted to confirm the result and identify more individual pesticides to cause rhinitis.

## Data availability

### Underlying data

Figshare: Dataset for study on pesticide exposure and rhinitis in Phitsanulok Thailand https://doi.org/10.6084/m9.figshare.14524326 ^22^.

This project contains the following underlying data:

• Dataset pesticide and rhinitis-Thailand (SAV and CSV).
• Data Dictionary (DOCX).

### Extended data

Figshare: Questionnaire-pesticide and rhinitis-Thailand. https://doi.org/10.6084/m9.figshare.14524335.v1 ^18^.

This project contains the following extended data:

• Questionnaire-pesticide and rhinitis-English (DOCX). (Study questionnaire in English.)
• Questionnaire-pesticide and rhinitis-Thai (DOCX). (Study questionnaire in Thai.)

Data are available under the terms of the Creative Commons Zero “No right reserved” data waiver (CC0 1.0 Public domain dedication).
