# Pesticide exposure and rhinitis: A cross-sectional study among farmers in Pitsanulok, Thailand

**DOI:** 10.12688/f1000research.53257.2

**Published:** 2023-05-26

**Authors:** Yuwayong Juntarawijit, Chudchawal Juntarawijit

**Affiliations:** 1 Faculty of Nursing, Naresuan University, Phitsanulok, 65000, Thailand; 2 Faculty of Agriculture, Natural Resources and Environment, Naresuan University, Phitsanulok, 65000, Thailand

**Keywords:** pesticide exposure, insecticide effect, herbicide effect, fungicide effect, rhinitis, farmer health

## Abstract

**Background**: Pesticide exposure has been suspected to cause rhinitis, a common disease that affects the health and well-being of millions of people around the world. This cross-sectional study aimed to examine the association between pesticide use and rhinitis prevalence among farmers in Phitsanulok province, Thailand.

**Methods:** Data on historical pesticide use and rhinitis were collected by an in-person interview questionnaire. Data from 9,649 participants were included in the analysis. The association between pesticide exposure and rhinitis was determined by multiple variable logistic regression, adjusted for potential confounding factors.

**Results:** The study found a strong association between pesticide exposure and the prevalence of rhinitis. The association was consistent across various types of pesticides (insecticides, herbicides, fungicides, rodenticides, and molluscicides) and individual pesticides. Some of the relationships were in a dose-response pattern. This finding was new as previous studies often reported the association of only a few specific pesticides.

**Conclusions:**The results from this large cross-sectional study strongly support existing literature on the potential effects of pesticides on rhinitis. In addition, the analysis showed that the rhinitis effect was in fact related to the properties of the types of pesticides rather than individual chemical toxicity. The impact of pesticides on rhinitis should receive more attention from public health and other organizations responsible for the farmers’ health.

## Background

Rhinitis is a common disease that affects general health, and quality of life. Approximately, 10% to 30% of the different populations worldwide suffer from this disease ^1^. One study in Bangkok, Thailand, has reported a prevalence of chronic rhinitis to be approximately 13% ^2^. In clinical terms, rhinitis refers to the inflammatory disease of the nasal mucosa, which can cause the following symptoms: nasal congestion, rhinorrhea, and sneezing ^3^. Although rhinitis does not have a strict classification criterion, it can be classified into allergic rhinitis (AR), and nonallergic rhinitis (NAR). Both have the same nasal symptoms, with the difference that AR is triggered by allergens. Several factors can trigger NAR, including cold air, climate change, cooking smells, chemical odour, cigarette smoke, volatile organic chemicals, exercise, alcohol ingestion ^4^, and cooking fumes ^5^. NAR can be further classified by their pathological mechanisms into several subtypes, including occupation rhinitis, hormonal rhinitis, drug-induced rhinitis, food-induced rhinitis, emotion-induced rhinitis, etc ^6^. Approximately 43% of all rhinitis cases are AR, and 23% are NAR, while 34% of the cases are a mixture of both ^4^.

Studies found pesticide exposure to increase the risk of several respiratory problems, e.g., asthma, chronic bronchitis ^7^, and rhinitis ^8^. A study among grape farmers in Greece reported a higher prevalence of AR among those who used pesticides ^9^. Another study in France found that children living in areas surrounding vineyards had a higher rate of rhinitis symptoms (OR=3.56; 95% CI 1.04–12.12) ^10^. In an occupational setting, a study found number of hours working in the greenhouse per day to be associated with rhinitis (OR, 1.85; 95% CI, 1.05–3.23) ^11^. A large survey of farmworkers in the United States of America (U.S.A) found that insecticide and herbicide use has significantly increased the risk of allergic rhinitis and asthma ^12^.

Currently, evidence on the effects of pesticides on rhinitis are limited. A recent systematic review by Rodrigues *et al.* ^13^ found pesticides exposure to associate with only asthma in children and adolescents but not allergic rhinitis. In addition, so far there were only a few individual pesticides identified as potential risk factors for rhinitis. Pesticides that were found to have a positive association with rhinitis were bipyridyl herbicides such as paraquat, and the broad-spectrum herbicides 2,4-D, glyphosate, dithiocarbamate fungicides including benomyl, and insecticide diazinon ^9, 14, 15^. It has been suggested that exposure to OP insecticides might exaggerated nasal glandular response resulting in increased rhinitis ^16^.

The main objective of this cross-sectional study was to determine the association between pesticide use and rhinitis among farmers in Phitsanulok, Thailand. The use of a large sample size in this study has provided the opportunity to assess also the risks that different groups and subgroups of pesticide exposure have had on these individuals. The study results would be useful for disease prevention, and comparison with other studies.

## Methods

### Study design and setting

This study was a cross-sectional study. Participants were farmers in Phitsanulok province, located about 370 km north of Bangkok, Thailand. In 2019, the province had approximately 865,368 people (342,787 households) from nine districts. The major crops in the province are rice, sugarcane, and maize ^17, 18^.

### Study participants and sampling procedure

Multistage sampling was used for the random selection of the participants. The three districts were randomly selected from nine districts in the Phitsanulok province. From all the selected districts, 18 out of 26 (69.2%) sub-districts were further selected. In each sub-district, all local hospitals participated in the study and provided support for data collection. In each sub-district, farmers were selected by village health volunteers (VHV), who were working in the hospitals. The data from the local authority and personal contacts were used by the VHV for selecting the farmers. Sample size (n = 9,649) was achieved with the snowball sampling technique. From each family, one adult aged 20 years or older who does agricultural work, was interviewed.

The minimum sample size was calculated to be 10,002, based on the following assumptions: significance level = 95%; power of detection = 80%; ratio of unexposed/exposed = 1; percent of unexposed with outcome = 10% ^2^; odds ratio = 1.2.

### Study questionnaire and data collection

Data were collected by using an in-person interview questionnaire (provided as Extended data in English and Thai) ^19^. Data on rhinitis was collected by using a modified form of SFAR (Score For Allergic Rhinitis) questionnaire which is recommended for population studies, where medical diagnosis and objective measurements were absent or difficult to obtain ^20^. The SFAR encompasses questions regarding the eight features of AR. Each of the items can be quantitatively scored and yield a maximum score of 16. AR refers to those with a score of 7 or more. Self-reported rhinitis was defined as the participant answering “yes” to the question: “during the last 12 months, have you ever had symptoms such as sneezing, or a runny, or blocked nose when you did not have a cold or the flu?”.

For pesticide exposure, the data was collected by a questionnaire used in our previous study ^21^. Data on the long-term use of pesticides, either by types of pesticides (insecticide, herbicide, fungicide, rodenticide, and molluscicide), or by specific individual pesticides, were collected. A list of 39 individual pesticides were chosen from those that were commonly used in Thailand and were reported to cause adverse health effects ^15, 21^. Participants were asked whether they have ever used pesticides, defined as a mixture, or spray pesticides, in their lifetime. Participants were also asked to provide data on the duration (days/year and total years) of pesticide use. This information was used to calculate total days, and quartiles of days using each pesticide in the farmers’ lifetime.

Lifetime pesticide use measured in days = [Number of days per year] × [total years]

The individual pesticides were six organochlorine insecticides (Aldrin, Chlordane, DDT, Dieldrin, Endosulfan, and Heptachlor); eleven organophosphate insecticides (Abamectin, Chlorpyrifos, Dicrotophos, Dichlorvos, EPN, Imidacloprid, Methamidophos, Mevinphos, Monocrotophos, Parathion/Folidol, and Profenofos); four carbamate insecticides (Carbaryl/sevin, Carbofuran, Carbosulfan, and Methomyl); and one pyrethroid insecticide Permethrin. The study also included eight herbicides (2,4 D, Acetrochlor, Alachlor, Ametryn, Butachlor, Diuron, Glyphosate, and Paraquat) and nine fungicides (Benomyl, Bordeaux mixture, Carbendazim, Copper sulphate, Mancozeb, Maneb, Metalaxyl, Propineb, and Thiophanate).

Data was collected from October 2020 to February 2021, by 210 VHV. Before data collection, these volunteers had to attend a one-day training program to be informed on the purpose of the study and to learn how to properly interview and collect data by using an online questionnaire. The interview mostly took place in the participant’s home. However, sometimes it was done in a local temple or hospital.

### Statistical analysis

Demographic data were analysed using descriptive statistics. Comparison of categorical data was analysed using the Chi-square test. The association between pesticide exposure and rhinitis prevalence was analysed using multivariable logistic regression, and both crude and adjusted odds ratios (OR) and 95% confidence interval (CI) were reported. Adjusted variables were gender (male, female), age (continuous), marital status (married, single, divorced/widow/separated), education (non-educated, primary school, secondary school, college degree or higher), family income (<5000 THB, 5001–10000 THB, 10001–3000 THB, >30000 THB), cigarette smoking (non-smoker, ex-smoker, current smoker), alcohol consumption (non- drinker, ex- drinker, regular- drinker).

To control a potential confounding effect of the use of other pesticides, correlation matrices of types and individual pesticides were developed. Those pesticides with highly correlation (Spearman coefficient ≥0.30) were identified and included the regression model. A list of potential confounding pesticides was presented in Table S1 and Table S2. The dose-response relationship was analysed using chi-squared tests for trend using an ordinal term for the quartile days as a category. This statistical analysis was available only when there was sufficient frequency of exposure and disease. Data analysis was performed using IBM SPSS version 26, and OpenEpi (online version 3.01). All statistical values were two-tailed, and a p-value <0.05, was considered as statistically significant.

### Ethical considerations

The study was approved by the Ethical Committee of Naresuan University (COA No. 657/2019) and written informed consent from the participants was obtained before the interview process.

## Results

It was found that the proportion of female participants was slightly higher than that of the male participants ( Table 1 and the underlying data ^22^). Most of these individuals were aged 40 years and older with an average age of 55 (±12 years), married (78.0%), finished primary school or with lower education (77.2), and had an average family income of 10,000 THB or less. A total of 8.7% of these females are cigarette smokers, and 13.7% consume alcohol.

**Table 1.** Demographic data of study participants.

Characteristics	Number (%), N = 9649
Gender	
Male	4163 (43.1)
Female	5486 (56.9)
Age, years	
20–30	306 (3.2)
31–40	840 (8.7)
41–50	2000 (20.7)
51–60	3174 (32.9)
61+	3329 (34.5)
Mean ± SD = 55 ± 12	
Min-Max = 20-92	
Marital status	
Married	7523 (78.0)
Single	737 (7.6)
Divorce/widow/separated	1389 (14.4)
Education	
Non-educated	402 (4.2)
Primary school	7046 (73.0)
Secondary school	2063 (21.4)
College degree or higher	138 (1.4)
Family income, THB/month	
<5000	3168 (32.8)
5001–10000	5739 (59.5)
10001–30000	668 (6.9)
>30000	74 (0.8)
Cigarette smoking	
Non-smoker	8511 (88.2)
Ex-smoker	303 (3.1)
Current smoker	835 (8.7)
Alcohol consumption	
Non-drinker	7865 (81.5)
Ex-drinker	461 (4.8)
Regular-drinker	1323 (13.7)

The prevalence of rhinitis was found to be 6.3% (609/9649) ( Table 2). Based on SFAR scoring, only about 36% of them had allergic rhinitis (SFAR score ≥7). Of the three symptoms, sneezing was found to be the most common (4.0%), followed by nasal congestion (3.0%), and runny nose (2.9%). Only 16.3% had eye irritations together with rhinitis symptoms. The months with the highest frequency of symptoms were March to July (summer season in Thailand), and November to February (winter season). The prevalence of rhinitis was significantly associated with marital status, education, family income, cigarette smoking, and alcohol consumption ( Table 3).

**Table 2.** Prevalence of rhinitis and its characteristics.

	N (%), N = 9649
Having symptoms in the past year	
Sneezing	390 (4.0)
Stuffy nose	294 (3.0)
Runny nose	282 (2.9)
Rhinitis (having one of the above three symptoms)	609 (6.3)
Types of rhinitis (N=411)	
Allergic rhnitis *	147 (35.8)
Non allergic rhnitis	264 (64.2)
SFAR score (N=411)	
1-3	49 (11.9)
3-6	215 (52.3)
7-9	107 (26.0)
10+	40 (9.7)
Having eyes irritation while having rhinitis	99 (16.3)
Month of having the symptoms (n=609)	
January	177 (29.3)
February	103 (17.0)
March	76 (12.6)
April	100 (16.5)
May	258 (42.6)
June	272 (44.9)
July	245 (40.5)
August	95 (15.7)
September	61 (10.1)
October	53 (8.8)
November	99 (16.4)
December	137 (22.6)
Allergens activated the symptoms (n=609)	
Dust	467 (76.7)
Mites	282 (46.3)
Smoke	275 (45.2)
Cooking fume	190 (31.4)
Straw or grass	101 (16.6)
Pet	33 (5.4)
Pollen	34 (5.6)
Patients who believed they have allergy	104 (17.1)
Patients that had allergic testing	36 (5.9)
Patients with positive result	24 (3.9)
Patients who were diagnosed with asthma or allergy	39 (6.7)

* Allergic rhinitis refers to those with SFAR core ≥7

**Table 3.** Association between demographic data and rhinitis prevalence.

	Not rhinitis	Rhinitis	P-value ^a^
Gender			0.176
Male	3884 (93.3)	279 (6.7)	
Female	5156 (94.0)	330 (6.0)	
Age			0.488
20–30	285 (93.1)	21 (6.9)	
31–40	777 (92.5)	63 (7.5)	
41–50	1867 (93.4)	133 (6.7)	
51–60	2983 (94.0)	191 (6.0)	
>60	3128 (94.0)	201 (6.0)	
Marital status			0.010 *
Married	7021 (93.3)	502 (6.7)	
Single	693 (94.0)	44 (6.0)	
Divorce/widow/separated	1326 (95.5)	63 (4.5)	
Education complete			<0.001 *
Non-educated	381 (94.8)	21 (5.2)	
Primary school	6655 (94.5)	391 (5.5)	
Secondary school	1877 (91.0)	186 (9.0)	
College degree or higher	127 (92.0)	11 (8.0)	
Family income, THB/month			<0.001 *
<5000	3067 (96.8)	101 (3.2)	
5001–10000	5330 (92.9)	409 (7.1)	
10001–30000	576 (86.2)	92 (13.8)	
>30000	67 (90.5)	7 (9.5)	
Cigarette smoking			0.001 *
Non-smoker	7997 (94.0)	514 (6.0)	
Ex-smoker	269 (88.8)	34 (11.2)	
Current smoker	774 (92.7)	61 (7.3)	
Alcohol consumption			<0.001 *
Non-drinker	7415 (94.3)	450 (5.7)	
Ex-drinker	406 (88.1)	55 (11.9)	
Regular-drinker	1219 (92.1)	104 (7.9)	

^a^ Chi-square test. * Statistically significant difference with p value <0.05

All five types of pesticides included in the study were found to be significantly related to rhinitis prevalence. Fungicides were significant until adjustments for using other pesticides were made, then it was shown to be not significant ( Table 4). Any pesticide use increased rhinitis risk by 1.79 folds (95% CI 1.09-2.94). The lowest OR value was 1.67 (95% CI 1.41-1.99) for fungicide and 7.19 (95% CI 4.67-11.06) for insecticide. Manyt of the associations were in a dose-response pattern. The association remained significant after adjusting for use of other types of pesticides.

**Table 4.** The associations between types of pesticides use and rhinitis prevalence.

Pesticide	Not rhinitis	Rhinitis	OR (Crude)	OR (Model1) ^a^	OR (Model2) ^b^
**Any pesticide**					
Yes	8575 (93.5)	592 (6.5)	**1.88 (1.15-3.07)** ^c^	**1.79 (1.09-2.94)**	NA
No	463 (96.5)	17 (3.5)	1.0	1.0	
**Insecticide**					
Yes	7044 (92.3)	587 (7.7)	**7.56 (4.93-11.61)**	**7.19 (4.67-11.06)**	**5.48 (3.47-8.67)**
No	1996 (98.9)	22 (1.1)	1.0	1.0	1.0
Q1	1837 (95.1)	94 (4.9)	4.64 (2.91-7.42)	4.22 (2.63-6.77)	3.33 (2.03-5.47)
Q2	2009 (93.7)	136 (6.3)	6.14 (3.90-9.68)	5.76 (3.64-9.11)	4.51 (2.78-7.32)
Q3	2048 (90.5)	215(9.5)	9.53 (6.12-14.83)	9.27 (5.92-14.49)	7.16 (4.45-11.54)
Q4	1150 (89.0)	142 (11.0)	11.20 (7.11-17.66)	11.31 (7.14-17.92)	8.85 (5.44-14.39)
P for trend		<0.001			
**Herbicide**					
Yes	7786 (93.0)	586 (7.0)	**4.74 (3.03-7.44)**	**4.25 (2.71-6.69)**	**3.64 (2.30-5.77)**
No	1254 (98.4)	20 (1.6)	1.0	1.0	1.0
Q1	2227 (95.2)	112 (4.8)	3.15 (1.95-5.10)	2.71 (1.67-4.41)	2.28 (1.40-3.74)
Q2	1864 (93.9)	122 (6.1)	4.10 (2.54-6.62)	3.65 (2.26-5.91)	3.09 (1.90-5.04)
Q3	2555 (92.0)	221 (8.0)	5.42 (3.42-8.61)	4.83 (3.03-7.70)	4.09 (2.55-6.57)
Q4	1140 (89.5)	134 (10.5)	7.37 (4.58-11.87)	6.98 (4.31-11.28)	5.99 (3.69-9.75)
P for trend		<0.001			
**Fungicide**					
Yes	3933 (91.8)	353 (8.2)	**1.79 (1.52-2.11)**	**1.67 (1.41-1.99)**	1.03 (0.83-1.27)
No	5107 (95.2)	256 (4.8)	1.0	1.0	1.0
Q1	1006 (93.7)	68 (6.3)	1.35 (1.02-1.78)	1.25 (0.94-1.66)	0.80 (0.59-1.09)
Q2	1020 (92.3)	100 (7.7)	1.66 (1.31-2.11)	1.57 (1.22-2.01)	1.01 (0.77-1.32)
Q3	766 (90.7)	79 (9.3)	2.06 (1.58-2.68)	1.94 (1.48-2.55)	1.14 (0.84-1.55)
Q4	959 (90.0)	106 (10.0)	2.21 (1.74-2.79)	2.03 (1.59-2.60)	1.22 (0.92-1.61)
P for trend		<0.001			
**Rodenticide**					
Yes	1669 (87.9)	230 (12.1)	**2.68 (2.26-3.18)**	**2.63 (2.20-3.14)**	**2.04 (1.63-2.55)**
No	7371 (95.1)	379 (4.9)	1.0	1.0	1.0
Q1	514 (88.9)	64 (11.1)	2.42 (1.83-3.20)	2.40 (1.80-3.20)	1.86 (1.35-2.56)
Q2	319 (83.5)	63 (16.5)	3.84 (2.88-5.13)	3.71 (2.76-5.01)	2.92 (2.11-4.04)
Q3	448 (90.3)	48 (9.7)	2.08 (1.52-2.86)	2.13 (1.54-2.94)	1.69 (1.20-2.39)
Q4	388 (87.6)	55 (12.4)	2.76 (2.04-3.72)	2.58 (1.90-3.52)	1.94 (1.38-2.74)
P for trend		<0.001			
**Molluscicide**					
Yes	1944 (889.1)	238 (10.9)	**2.34 (1.98-2.78)**	**2.29 (1.92-2.73)**	NA
No	7096 (95.0)	371 (5.0)	1.0	1.0	
Q1	573 (86.6)	89 (13.4)	2.97 (2.32-3.80)	2.98 (2.31-3.85)	NA
Q2	404 (87.6)	57 (12.4)	2.70 (2.01-3.63)	2.62 (1.93-3.55)	NA
Q3	544 (92.5)	44 (7.5)	1.55 (1.12-2.14)	1.51 (1.08-2.10)	NA
Q4	423 (89.8)	48 (10.2)	2.17 (1.58-2.98)	2.06 (1.49-2.85)	NA
P for trend		<0.001			

^a^ Model1: Adjusted variables were gender (male, female), age (continuous), marital status (married, single, divorce/willow/separated), education (non-educated, primary school, secondary school, college degree or higher), family income (<5000 THB, 5001-10000 THB, 10001-30000 THB, >30000 THB), cigarette smoking (non-smoker, ex-smoker, current smoker), alcohol consumption (non-drinker, ex-drinker, regular-drinker). ^b^ Model2: Adjusted for all factors in the model1 and using other types of pesticide. ^c^ Significant OR were indicated in bold numbers.

For individual pesticides, the study found 32 out of 39 to be significantly associated with rhinitis after adjusted for demographic factors. Those pesticides were six herbicides, nine organophosphates (OP) insecticides, four carbamate insecticides, one pyrethroid insecticide, five OC insecticides, and seven fungicides ( Table 5). Most of the OR decreased but remained significant after the adjustment for using other potential confounding pesticides. The association of some pesticides were in a dose-response pattern ( Table 6).

**Table 5.** The associations between individual pesticide and rhinitis prevalence.

	Not rhinitis	Rhinitis	OR (Crude)	OR (Model1) ^a^	OR (Model2) ^b^
**Insecticides**					
**Organochlorine**					
Aldrin	6.1	29.4	6.36 (3.75-10.79)	4.83 (2.78-8.39)	2.02 (0.97-4.19)
Chlordane	6.1	20.0	3.82 (2.40-6.07)	3.43 (2.11-5.58)	1.95 (1.07-3.55)
Dieldrin	6.1	28.2	6.04 (3.74-9.76)	5.15 (3.11-8.53)	2.70 (1.41-5.14)
DDT	5.6	23.2	5.05 (3.91-6.55)	4.57 (3.43-5.99)	NA
Endosulfan	5.4	11.7	2.31 (1.91-2.79)	2.17 (1.78-2.64)	1.60 (1.26-2.03)
Heptachlor	6.1	20.2	3.87 (2.47-6.04)	3.29 (2.07-5.24)	1.83 (1.02-3.29)
**Organophosphate**					
Abamectin	6.8	5.9	0.85 (0.73-1.01)	0.82 (0.69-0.97)	0.68 (0.56-0.81)
Chlorpyrifos	5.5	9.0	1.71 (1.43-2.04)	1.53 (1.28-1.84)	1.08 (0.85-1.37)
Dicrotophos	6.2	11.3	1.93 (1.23-3.04)	1.70 (1.07-2.71)	0.93 (0.52-1.63)
Dichlorvos	6.2	16.8	3.06 (1.83-5.12)	2.77 (1.63-4.70)	2.57 (1.40-4.71)
EPN	5.8	24.0	5.11 (3.81-6.86)	4.15 (3.06-5.62)	NA
Imidacloprid	6.1	11.9	2.08 (1.52-2.85)	1.88 (1.36-2.61)	1.55 (1.09-2.20)
Methamidophos	6.0	12.8	2.30 (1.71-3.08)	2.06 (1.522-2.79)	NA
Mevinphos	6.2	14.9	2.64 (1.59-4.39)	2.32 (1.36-3.94)	2.04 (1.14-3.62)
Monocrotophos	6.1	15.6	2.85 (1.96-4.13)	2.46 (1.66-3.63)	2.31 (1.52-3.52)
Parathion/ Folidol	4.7	17.6	4.37 (3.66-5.22)	3.95 (3.29-4.74)	NA
Profenofos	6.3	8.0	1.30 (0.81-2.10)	1.30 (0.80-2.12)	0.96 (0.57-1.65)
**Carbamate**					
Carbaryl/ Sevin	5.7	13.6	2.58 (2.05-3.23)	2.31 (1.82-2.93)	NA
Carbosulfan	5.6	11.5	2.21 (1.81-2.69)	2.09 (1.70-2.56)	1.44 (1.13-1.85)
Carbofuran	5.6	13.7	2.67 (2.14-3.32)	2.34 (1.86-2.93)	1.84 (1.44-2.35)
Methomyl	5.9	12.7	2.31 (1.76-3.03)	2.02 (1.53-2.68)	NA
**Pyrethroid**					
Permethrin	6.0	8.9	1.54 (1.23-1.94)	1.60 (1.27-2.03)	1.15 (0.88-1.49)
**Herbicides**					
2,4-D	5.1	7.4	1.48 (1.25-1.75)	1.38 (1.61-1.63)	NA
Acetrochlor	6.3	6.3	0.99 (0.59-1.65)	0.96 (0.57-1.62)	NA
Alachlor	6.1	8.2	1.37 (1.06-1.77)	1.30 (1.00-1.69)	1.47 (1.08-2.03)
Ametryn	6.3	7.0	1.13 (0.69-1.83)	0.94 (0.57-1.54)	NA
Butachlor	5.2	9.8	1.99 (1.68-2.36)	1.82 (1.52-2.17)	1.44 (1.17-1.77)
Diuron	6.2	12.8	2.21 (1.30-3.76)	1.73 (1.00-2.99)	2.33 (1.14-4.76)
Glyphosate	2.7	6.8	2.59 (1.80-3.74)	2.43 (1.68-3.52)	NA
Paraquat	4.4	7.5	1.78 (1.48-2.14)	1.54 (1.28-1.87)	NA
**Fungicide**					
Benomyl	6.2	13.3	2.32 (1.42-3.78)	2.03 (1.22-3.36)	NA
Bordeaux mixture	6.2	29.0	6.23 (34.58-10.84)	5.67 (3.16-10.16)	3.44 (1.78-6.65)
Carbendazim	5.8	9.5	1.71 (1.40-2.11)	1.57 (1.27-1.94)	1.43 (1.14-1.79)
Copper sulfate	6.3	6.1	0.97 (0.68-1.38)	0.82 (0.57-1.17)	NA
Mancozeb	5.9	11.7	2.13 (1.67-2.71)	1.99 (1.55-2.55)	NA
Maneb	6.3	7.6	1.23 (0.86-1.76)	1.24 (0.86-1.80)	NA
Metalaxyl	6.1	8.2	1.37 (1.05-1.79)	1.35 (1.03-1.78)	NA
Propineb	6.1	11.0	1.90 (1.40-2.59)	1.84 (1.34-2.53)	NA
Thiophanate	6.2	11.3	1.93 (1.28-2.91)	1.90 (1.25-2.90)	NA

^a^ Model1: Adjusted variables were gender (male, female), age (continuous), marital status (married, single, divorce/willow/separated), education (non-educated, primary school, secondary school, college degree or higher), family income (<5,000 THB, 5,001-10,000 THB, 10,001-30,000 THB, >30,000 THB), cigarette smoking (non-smoker, ex-smoker, current smoker), alcohol consumption (non-drinker, ex-drinker, regular-drinker). ^b^ Model2: Adjusting for all the factors in the model1 and using of other pesticides.

**Table 6.** Dose-response relationship of selected individual pesticides.

Pesticide	Exposure	Rhinitis	OR	P for trend *
**Herbicide**				
2,4-D	Not use	5.1	1.0	0.01
	Q1	7.7	1.40 (1.10-1.78)	
	Q2	8.5	1.60 (1.26-2.03)	
	Q3	7.6	1.42 (1.10-1.83)	
	Q4	5.6	1.07 (.80-1.42)	
Alachlor	Not use	6.1	1.0	0.01
	Q1	7.2	1.10 (.69-1.76)	
	Q2	12.5	2.08 (1.29-3.35)	
	Q3	4.7	0.76 (.45-1.30)	
	Q4	13.4	2.11 (1.24-3.59)	
Butachlor	Not use	5.2	1.0	<0.001
	Q1	9.7	1.85 (1.38-2.48)	
	Q2	9.9	1.94 (1.47-2.55)	
	Q3	10.1	1.80 (1.34-2.42)	
	Q4	9.2	1.63 (1.16-2.27)	
Glyphosate	Not use	2.7	1.0	<0.001
	Q1	5.4	1.84 (1.23-2.74)	
	Q2	6.5	2.35 (1.57-3.53)	
	Q3	8.4	3.07 (2.07-4.55)	
	Q4	7.3	2.69 (1.80-4.03)	
Paraquat	Not use	4.4	1.0	<0.001
	Q1	6.6	1.28 (1.00-1.64)	
	Q2	6.9	1.43 (1.09-1.87)	
	Q3	9.6	2.04 (1.61-2.60)	
	Q4	7.2	1.51 (1.16-1.97)	
**Organophosphate**				
Monocrotophos	Not use		1.0	<0.001
	Q1	11.9	1.80 (.80-4.07)	
	Q2	15.5	2.51 (1.20-5.25)	
	Q3	3.4	0.49 (.12-2.05)	
	Q4	38.1	7.57 (3.88-14.77)	
Parathion/Folidol	Not use	4.7	1.0	<0.001
	Q1	10.8	2.19 (1.52-3.16)	
	Q2	13.2	2.92 (2.06-4.13)	
	Q3	20.6	4.70 (3.44-6.42)	
	Q4	26.8	6.91 (5.21-9.18)	
**Carbamate**				
Carbaryl/Sevin	Not use	5.7	1.0	<0.001
	Q1	10.1	1.63 (1.0-2.66)	
	Q2	13.8	2.46 (1.57-3.87)	
	Q3	11.9	1.84 (1.16-2.90)	
	Q4	19.3	3.73 (2.48-5.63)	
**Organochlorine**				
DDT	Not use	5.6	1.0	<0.001
	Q1	21.3	3.70 (2.20-6.24)	
	Q2	21.1	4.72 (2.91- 7.66)	
	Q3	16.3	3.07 (1.78-5.29)	
	Q4	42.4	9.50 (5.47-16.50)	
Endosulfan	Not use	5.4	1.0	<0.001
	Q1	15.6	2.93 (2.21-3.89)	
	Q2	6.5	1.23 (0.78-1.95)	
	Q3	11.5	2.06 (1.41-3.00)	
	Q4	11.4	2.20 (1.53-3.17)	
**Fungicide**				
Carbendazim	Not use	5.8	1.0	<0.001
	Q1	12.0	1.94 (1.38-2.73)	
	Q2	8.4	1.50 (1.01-2.21)	
	Q3	10.4	1.70 (1.18-2.45)	
	Q4	6.5	0.98 (0.58-1.66)	
Metalaxyl	Not use	6.1	1.0	<0.001
	Q1	7.1	1.18 (0.70-2.01)	
	Q2	4.6	0.72 (0.37-1.43)	
	Q3	7.7	1.23 (0.74-2.04)	
	Q4	15.6	2.80 (1.75-4.49)	
Propineb	Not use	6.1	1.0	<0.001
	Q1	18.4	1.05 (0.52-2.09)	
	Q2	11.1	1.76 (0.89-3.46)	
	Q3	12.1	2.27 (1.29-3.98)	
	Q4	15.2	2.55 (1.44-4.54)	
Thiophanate	Not use	6.2	1.0	<0.001
	Q1	13.3	2.07 (0.97-4.44)	
	Q2	4.8	0.66 (0.20-2.15)	
	Q3	8.6	1.71 (0.67-4.39)	
	Q4	19.0	3.68 (1.84-7.34)	

* Chi-squared tests for trend

## Discussion

The study found a strong association between the history of pesticide exposure and the prevalence of rhinitis. The association was consistent across various types of pesticides, including insecticides, herbicides, fungicides, rodenticides, and molluscicide ( Table 4). For individual pesticides, 32 out of 39 showed a significant relation with the disease and some with a dose-response pattern ( Table 5, Table 6). The relationship exists after adjustment by potential confounding factors including the use of other pesticides. This finding was new as previous studies often reported the association of only a few specific pesticides. In addition, the OR in this study was higher. For instance, a study among grape farmers in Crete, Greece reported the highest risks for a lifetime exposure to paraquat and other bipyridyl herbicide, (OR, 2.2; 95% CI, 1.0 to 4.8), dithiocarbamate fungicides (OR, 2.5; 95% CI, 1.1 to 5.3) and carbamate insecticides (OR, 3.0; 95% CI, 1.4 to 6.5) ^9^. In Agricultural Health Study, an elevated risk of rhinitis was observed among those who had a previous year exposure to herbicides (glyphosate, petroleum oil, 2,4-D), organophosphates insecticide (chlorpyrifos, diazinon, dichlorvos, malathion), carbarmate insecticide (carbaryl/savin, carbofuran), fungicide (benomyl, captan) ^15, 16^.

The difference might be explained by the fact that most of the rhinitis cases were NAR and the exposure was chronic. The study results also implied that the rhinitic effect might be related to the general characteristics of pesticide types, rather than individual properties. Pesticides can affect AR and NAR by several biomechanisms, involving immune and nonimmune pathways, after acute and chronic exposure. For acute high dose exposure, pesticides and the solvent composition of the mixture that contains them can directly irritate the nose and throat ^23^. Organophosphate and carbamate can cause cholinergic stimulation of the nasal mucosa and inhibit acetylcholinesterase thus causing more secretion of mucus in the nose and airway system ^24^. Organochlorines and pyrethroids are another type of insecticides designed to target the nervous system of insects. Exposure to the compounds can cause hyperexcitability of nerve cells by interacting with the sodium channel and keeping it open longer ^25^. Pyrethroid like other insecticides can irritate the respiratory tract, nose, and throat ^26^. For herbicides, evidence from both animal and human studies showed that it can cause airway inflammation ^27^. An experimental study found that acute exposure to herbicide 2,4-D increases the mast cells in the nasal epithelium of mice ^28^. Paraquat was another herbicide with high toxicity to the respiratory system, and previous studies reported a positive association of it and several respiratory illnesses, including rhinitis ^9^ and wheezing ^29^. In an experimental study, it was found that fungicides have cytotoxic effects on bronchial epithelial cells ^30^.

For chronic exposure, pesticide exposure can lead to exaggerated responses that increase the risk of rhinitis ^16^. This continual response had been involved in the development of other chronic diseases, e.g., asthma, and bronchitis ^31^. In a respiratory health study, OPs affect the bronchial lining and increase susceptibility to allergens or other stimuli ^32^. OP can also induce airway hyperreactivity at doses lower than those required to cause acetylcholinesterase inhibition ^33, 34^.

Although it is hard to differentiate between AR and NAR, by using the SFAR questionnaire and its scoring method ^20^, the study found that most of rhinitis cases in this study (64.2%) were NAR ( Table 2). Further analysis by comparing the association of pesticides and types of rhinitis revealed that NAR had a stronger association ( Table S3). This piece of information was often missed in the literature as most previous studies on rhinitis did not have enough data or focus on allergic types. The information helps explain why the results OR found in this study were higher than those previously reported.

This study has some limitations. The study did not have information and control for other potential confounding pollutants, such as grain and hay handling, and maintenance activities, e.g., repairing engines and pesticide equipment ^15^. It was also very likely that participants were exposed to environmental pesticides. However, it is more likely that both the control and study groups will have a similar risk to those factors and the problems would bias toward the null. The information on pesticide exposure was dependent solely on the questionnaire method. However, as the study is focusing on long-term exposure, the questionnaire method was the best option ^35^. This type of study could also be subject to recall bias as the case groups might be more aware of pesticide use than the control. However, the problem was less likely to occur because the information on the rhinitic effect of pesticides was not yet available in Thailand. In addition, the bias would only minimize but not increase the association ^36^. By using a cross-sectional design, the cause-effect association cannot be directly determined.

## Conclusion

The results from this large cross-sectional study strongly support exiting literature on the potential effects of chronic exposure to pesticides on rhinitis. The study was the first to confirm that most individual pesticides from various groups are associated with rhinitis, especially the NAR type. The effects of pesticides on rhinitis should receive more public attention, and the information be incorporated into the disease prevention program.

## Figures and Tables

**Table S1.** Correlation matrix for types of pesticides.

		1	2	3	4	5
1	Insecticides	1.00	.50	.42	.24	.26
2	Herbicides		1.00	.30	.17	.19
3	Fungicides			1.00	.48	.46
4	Rodenticides				1.00	.55
5	Molluscicides					1.00

**Table S2.** Correlation matrix of individual pesticides.

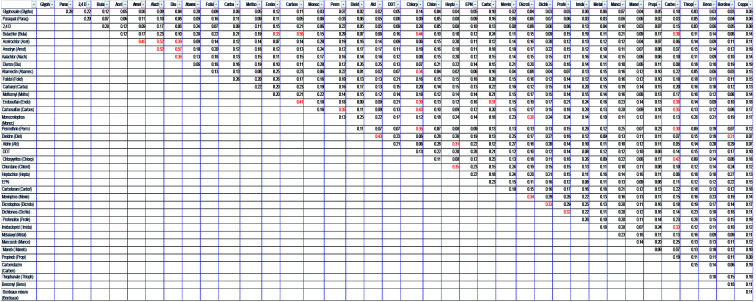

**Table S3.** The odds ratio of AR and NAR.

Pesticide	Rhinitis (Combinded)	AR	NAR
Aldrin	4.83 (2.78-8.39)	1.04 (0.25-4.42)	9.64 (5.32-17.46)
Endosulfan	2.17 (1.78-2.64)	3.54 (2.51-4.99)	2.90 (2.21-3.79)
Chlorpyrifos	1.53 (1.28-1.84)	2.17 (1.55-3.05)	2.46 (1.91-3.17)
Parathion/ Folidol	3.95 (3.29-4.74)	2.76 (1.93-3.96)	2.62 (1.99-3.46)
Carbaryl/Sevin	2.31 (1.82-2.93)	2.40 (1.56-3.70)	2.95 (2.15-4.04)
Permethrin	1.60 (1.27-2.03)	1.17 (0.72-1.90)	2.80 (2.09-3.76)
2,4-D	1.38 (1.61-1.63)	2.68 (1.83-3.91)	3.35 (2.48-4.54)
Butachlor	1.82 (1.52-2.17)	1.99 (1.42-2.80)	3.45 (2.67-4.45)
Glyphosate	2.43 (1.68-3.52)	2.34 (1.14-4.83)	4.63 (2.17-9.87)
Paraquat	1.54 (1.28-1.87)	2.46 (1.62-3.74)	1.47 (1.11-1.94)
Bordeaux mixture	5.67 (3.16-10.16)	4.29 (1.62-11.36)	5.94 (2.94-12.01)
Thiophanate	1.90 (1.25-2.90)	2.49 (1.28-4.86)	2.25 (1.30-3.90)
Mean±SD		2.44±0.85	3.71±2.10
		t-test, p-value = 0.006 ; N=12	

## Data Availability

Figshare: Dataset for study on pesticide exposure and rhinitis in Phitsanulok Thailand https://doi.org/10.6084/m9.figshare.14524326 ^22^. This project contains the following underlying data: Dataset pesticide and rhinitis-Thailand (SAV and CSV). Data Dictionary (DOCX). Figshare: Questionnaire-pesticide and rhinitis-Thailand. https://doi.org/10.6084/m9.figshare.14524335.v1 ^19^. This project contains the following extended data: Questionnaire-pesticide and rhinitis-English (DOCX). (Study questionnaire in English.) Questionnaire-pesticide and rhinitis-Thai (DOCX). (Study questionnaire in Thai.) Data are available under the terms of the Creative Commons Zero “No right reserved” data waiver (CC0 1.0 Public domain dedication).
