# Supplementary material for: Pesticide exposure and rhinitis: A cross-sectional study among farmers in Pitsanulok, Thailand
Source: F1000Res. 2023 Dec 5;10:474. Originally published 2021 Jun 15. [Version 3] doi: 10.12688/f1000research.53257.3 (PMC11369590; doi:10.12688/f1000research.53257.3)
Supplement: Peer review history

# Reviewer report: Reviewer response for version 3

**DOI:** 10.5256/f1000research.159653.r227986

**Published:** 2024-09-02

**Authors:** Bente Elisabeth Moen

**Affiliations:** 1 University of Bergen, Bergen, Norway

The authors have revised the manuscript nicely.

 However,  it is recommended to delete the second sentence of the Conclusions in the Abstract - it is difficult to understand and it is not in the conclusion at the end of the discussion.

Is the work clearly and accurately presented and does it cite the current literature?

Yes

If applicable, is the statistical analysis and its interpretation appropriate?

Partly

Are all the source data underlying the results available to ensure full reproducibility?

Partly

Is the study design appropriate and is the work technically sound?

Partly

Are the conclusions drawn adequately supported by the results?

Partly

Are sufficient details of methods and analysis provided to allow replication by others?

Partly

Reviewer Expertise:

Occupational health and neurotoxicology

I confirm that I have read this submission and believe that I have an appropriate level of expertise to confirm that it is of an acceptable scientific standard.

# Reviewer report: Reviewer response for version 2

**DOI:** 10.5256/f1000research.148334.r217833

**Published:** 2023-11-21

**Authors:** Patricia Segura-Medina

**Affiliations:** 1 Instituto Nacional de Enfermedades Respiratorias Ismael Cosío Villegas, Calzada de Tlalpan 4502, CP, Mexico

Is an Interesting study large based on a cross-sectional study aimed to determine the association between pesticide use and rhinitis prevalence among farmers.

 Data were obtained form data bases of pesticide use and correlated with rhinitis reported cases by interview questionnaire. Data from 9,649 participants were included in the analysis. The association between pesticide exposure and rhinitis was determined by multiple variable logistic regression, adjusted for potential confounding factors.

 Results are interesting, as well as the associations founded is a Local study that can be applied for other latitudes.

Is the work clearly and accurately presented and does it cite the current literature?

Yes

If applicable, is the statistical analysis and its interpretation appropriate?

Yes

Are all the source data underlying the results available to ensure full reproducibility?

Yes

Is the study design appropriate and is the work technically sound?

Yes

Are the conclusions drawn adequately supported by the results?

Yes

Are sufficient details of methods and analysis provided to allow replication by others?

Yes

Reviewer Expertise:

Asthma, COPD, Air pollution and health effects, Organophosphates and Asthma, Respiratory Toxicology

I confirm that I have read this submission and believe that I have an appropriate level of expertise to confirm that it is of an acceptable scientific standard.

# Response

**Published:** 2023-12-01

**Authors:** Chudchawal Juntarawijit

**Affiliations:** Naresuan University, Thailand

Thank you to the reviewer for approving the paper. Your insights and feedback have been invaluable.

# Reviewer report: Reviewer response for version 2

**DOI:** 10.5256/f1000research.148334.r212014

**Published:** 2023-10-13

**Authors:** Bente Elisabeth Moen

**Affiliations:** 1 University of Bergen, Bergen, Norway

This is an interesting study about rhinitis and pesticide exposure. It is a cross-sectional study among 9 649 Thailand farmers, interviewed about rhinitis and pesticide exposure. Regression analyses show associations between the exposure to different pesticides and the rhinitis.

 The manuscript is well written and easy to understand. However, there are several methodological challenges in the study:

- There are no clear hypotheses in the study. Pesticides consist of many substances, and they are from different chemical classes. There is no logic in all of these to be causing rhinitis. Rhinitis might be caused by allergens or irritants, and the authors should have commented on this in the introduction and tried to make specific hypotheses related to specific pesticides. When this is not done, it should be added as a discussion point in the Discussion.
- The selection of workers is not totally clear. How were the farmers addressed? How did the researchers meet them, and how was the circumstances when the interviews were conducted? Did they answer in a group of people, or in rooms where no one heard the answer. At work or at home? This should be described and the influence of the situation should be discussed in the Discussion.
  The snowball sampling must be described in detail, how did this happen?
- The sample calculations described must have references to where the figures came from.
- The interview was based on a questionnaire that originally was French, and the different cultures in France and Thailand should be mentioned as a challenge in the Discussion.
- Workers seldom know a lot about pesticides they used. How could the workers know exactly which pesticides they had been exposed to? This part of the interview should have been described better.
- In the Discussion, the authors must add text on discussion of 2 important weaknesses:
  1. Common method bias: This study collected data on the outcome rhinitis at the same time as exposure information (if understood correctly, it is not totally clear described). This might cause a bias, and make workers report what they think is the situation, more than what is true.
  2. Multiple testing. Very many statistical tests have been performed, and this makes the likelihood of having significant results high. Testing should have been done with corrections, adjusting p-values derived from these tests, to avoid false positive results.
  At least this should be clearly addressed in the discussion.
- When all these methodological weaknesses exist in a study, the authors need to revise the conclusions. The results are far from clear due to these weaknesses.
  At the start of the discussion, the text is: *The study found a strong association between the history of pesticide exposure and the prevalence of rhinitis.* 
  This must be changed to: *The study shows an association between the history of pesticide exposure and rhinitis. However, the results must be interpreted with caution due to several methodological weaknesses.*
  In conclusion: - remove the word ‘ strongly’ in the first sentence. The next sentence should be deleted: *The study was the first to confirm that most individual pesticides from various groups are associated with rhinitis, especially the NAR type.*
  These details are not likely to be true.
- The discussion should add more about why it is important to study rhinitis. This symptom/disease might be the start of a more serious health problem, asthma for instance, and by observing the existence of rhinitis, one can prevent more serious health problems. This is the most important message from this study and must be highlighted and not forgotten.

Is the work clearly and accurately presented and does it cite the current literature?

Yes

If applicable, is the statistical analysis and its interpretation appropriate?

Partly

Are all the source data underlying the results available to ensure full reproducibility?

Partly

Is the study design appropriate and is the work technically sound?

Partly

Are the conclusions drawn adequately supported by the results?

Partly

Are sufficient details of methods and analysis provided to allow replication by others?

Partly

Reviewer Expertise:

Occupational health and neurotoxicology

I confirm that I have read this submission and believe that I have an appropriate level of expertise to confirm that it is of an acceptable scientific standard, however I have significant reservations, as outlined above.

# Response

**Published:** 2023-12-01

**Authors:** Chudchawal Juntarawijit

**Affiliations:** Naresuan University, Thailand

**Comments and suggestions**

**Comment:**

 This is an interesting study about rhinitis and pesticide exposure. It is a cross-sectional study among 9 649 Thailand farmers, interviewed about rhinitis and pesticide exposure. Regression analyses show associations between the exposure to different pesticides and the rhinitis.

 The manuscript is well written and easy to understand. However, there are several methodological challenges in the study:

- There are no clear hypotheses in the study. Pesticides consist of many substances, and they are from different chemical classes. There is no logic in all of these to be causing rhinitis. Rhinitis might be caused by allergens or irritants, and the authors should have commented on this in the introduction and tried to make specific hypotheses related to specific pesticides. When this is not done, it should be added as a discussion point in the Discussion.

**Response:**

 We appreciate your thoughtful comments and suggestions. They are very useful, and we hope we do enough to address the issues and to improve the paper’s quality.

 Yes, I agree that rhinitis might be caused by any allergens or irritants, and there is no logic to believe that all pesticides will equally affect rhinitis. As you know, with limited literature, there was not enough data to hypothesize on the association between specific pesticide and rhinitis. Therefore, our main objective was to affirm the results of previous studies and to explore some more specific potential predictive pesticides. Another point was that the effects of allergens and irritants as potential confounders has been further discussed, and more information has been added to the Introduction as suggested.

**Comment:**

 The selection of workers is not totally clear. How were the farmers addressed? How did the researchers meet them, and how were the circumstances when the interviews were conducted? Did they answer in a group of people, or in rooms where no one heard the answer. At work or at home? This should be described, and the influence of the situation should be discussed in the Discussion.

 The snowball sampling must be described in detail, how did this happen?

**Response:**

 In this study, we use village health volunteers (VHV) to collect the data. The VHVs work as public health officers and they normally know every household in their community. The process begins with the VHV contacting the target household and visiting the household to conduct the interview. After finishing, they moved on to the next house and repeated this process until the target number was reached. We did not allow group interviews so there would not be a problem where interviewees influence each other. Sorry for not being aware of the issue and not clearly presenting the information. More information on sampling has been added in the Methods section. Since a large number of villagers participated in the study, so there were no concerns about the representation of the group.

**Comment:**

 The sample calculations described must have references to where the figures came from.

**Response:**

 The relevant references have been added.

**Comment:**

 The interview was based on a questionnaire that originally was French, and the different cultures in France and Thailand should be mentioned as a challenge in the Discussion.

**Response:**

 Although, the original was in French, most of the questions are simple yes or no questions, and asking a common symptom such as, sneezing, runny, and blocked nose. The statement has been added to the discussion as suggested.  

**Comment:**

 Workers seldom know a lot about pesticides they use. How could the workers know exactly which pesticides they had been exposed to? This part of the interview should have been described better.

**Response:**

 Thank you for mentioning the issue. That was exactly what happened and we tried to minimize the problem by including all the names of each specific pesticide, especially commercial names in the question and read all of them to the workers when interviewed. We expected that this action could minimize recall bias to some extent. The information has been added to the Methods.

**Comment:**

 In the Discussion, the authors must add text on discussion of 2 important weaknesses:

 1. Common method bias: This study collected data on the outcome of rhinitis at the same time as exposure to the information (if understood correctly, it is not totally clear as described). This might cause a bias, and make workers report what they think is the situation, more than what is true.

**Response:**

 Thank you for the insightful suggestions and the two biases have been acknowledged in the study limitations.

 In this study, data on pesticides used in the past and the data on rhinitis were collected at the same visit and using the same questionnaire method for self-report data collection. This could cause common method bias and affect the validity of the association of the two variables. To minimize the bias, first we use a face-to-face interview instead of a self-administer questionnaire. The survey questionnaire was designed so that exposure information was collected before some health effects and rhinitis. In addition, as the information is rather new, the workers were unlikely to be aware of the relationship between pesticide exposure and rhinitis.

**Comment:**

 2. Multiple testing. Very many statistical tests have been performed, and this makes the likelihood of having significant results high. Testing should have been done with corrections, adjusting p-values derived from these tests, to avoid false positive results.

 At least this should be clearly addressed in the discussion.

**Response:**

 Yes, we agree that the results might subject to the problem of multiple testing as several individual pesticides were tested against the disease. To acknowledge it, a statement has been added to the study limitations.

**Comment:**

 When all these methodological weaknesses exist in a study, the authors need to revise the conclusions. The results are far from clear due to these weaknesses.

 At the start of the discussion, the text is: *The study found a strong association between the history of pesticide exposure and the prevalence of rhinitis.* 

 This must be changed to: *The study shows an association between the history of pesticide exposure and rhinitis. However, the results must be interpreted with caution due to several methodological weaknesses.*

 In conclusion: - remove the word ‘strongly’ in the first sentence. The next sentence should be deleted: *The study was the first to confirm that most individual pesticides from various groups are associated with rhinitis, especially the NAR type.*

 These details are not likely to be true.

**Response:**

 Thank you very much for your kind suggestions. All of them are accepted and the statements have been revised as suggested.

****

**Comment:**

 The discussion should add more about why it is important to study rhinitis. This symptom/disease might be the start of a more serious health problem such as asthma, and by observing the existence of rhinitis, one can prevent more serious health problems. This is the most important message from this study and must be highlighted and not forgotten.

**Response:**

 More information on the study's strength and importance of study rhinitis have been added to the discussion.
